# Implementation of Virtual Reality in Health Professional Higher Education: Protocol for a Scoping Review

**DOI:** 10.2196/37222

**Published:** 2022-07-05

**Authors:** Silje Stangeland Lie, Nikolina Helle, Nina Vahl Sletteland, Miriam Dubland Vikman, Tore Bonsaksen

**Affiliations:** 1 Faculty of Health Studies VID Specialized University Stavanger Norway; 2 Faculty of Health Studies VID Specialized University Bergen Norway; 3 Department of Health and Nursing Sciences Faculty of Social and Health Sciences Inland Norway University of Applied Sciences Elverum Norway

**Keywords:** virtual reality, higher education, medical education, health care professional education, continuing education, implementation, technology, scoping review, Google Scholar, health professional

## Abstract

**Background:**

The use of virtual reality in higher education show great potential to promote novel and innovative learning experiences. Until recently, virtual reality has mostly been used in technical higher education, but lately medical education programs have begun using virtual reality. Virtual reality for health professional education improves the knowledge and skills of health professionals compared with traditional or other digital education initiatives. However, the implementation of technology in higher education is slow because of barriers to technology use and innovative and successful practices are not shared. It is, therefore, of great interest to explore how virtual reality is implemented in higher health professional and continuing education.

**Objective:**

The aim of this scoping review is to identify studies that reported implementation of virtual reality in higher health professional education, to identify barriers and facilitators for implementation, and to highlight research gaps in this area.

**Methods:**

The scoping review will be conducted according to JBI Evidence Synthesis methodologies. CINAHL, the Academic Search Elite and Education Source electronic databases, and Google Scholar will be searched for studies published between 2017 and 2022. In addition, manual searching of key items, reference tracking, and citation tracking will be performed. Searches for white papers will also be manually conducted. All authors will independently extract data from full-text papers. We will use qualitative content analysis to abstract the findings.

**Results:**

The literature searches were conducted in January and February 2022. The review is expected to be completed by fall 2022, after which time it will be submitted for publication.

**Conclusions:**

We anticipate that, from the review, we will be able to coordinate recommendations for and present the challenges of virtual reality initiatives in health professional education programs. We will present recommendations for future research.

**International Registered Report Identifier (IRRID):**

DERR1-10.2196/37222

## Introduction

### Background

Virtual reality is defined as a digital representation of a 3D environment [1]. Immersive virtual reality, wherein head-mounted displays are used to block out the real world, is now the general understanding of what constitutes virtual reality [2]. In higher education, the use of virtual reality shows great potential to promote novel and innovative learning experiences [3]. Virtual reality offers students and health care professionals a platform with which they can experience and learn how to master situations without putting patients or themselves in any risk of harm [4]. Until recently virtual reality has mostly been used in technical higher education programs, such as engineering, computer science, and astronomy [5], but lately, there has been growth in interest and the use of virtual reality in medical education programs [6]. A review [7] on virtual reality for health professional education found that, in comparison with traditional or other digital education initiatives, virtual reality initiatives improved health professionals’ knowledge and skills [7]. However, the implementation of technology in higher education is slow because of barriers to technology use and innovative and successful practices are not shared [8].

### Preliminary Search and Review

Preliminary searches of PROSPERO, the Cochrane Database of Systematic Reviews, JBI Evidence Synthesis, and the Journal of Medical Internet Research, using the search string “implement* virtual reality” were conducted to gain an overview of in-progress reviews. No current or in-progress scoping reviews or systematic reviews on the implementation of virtual reality in health professional education were identified; however, a protocol [9] for a scoping review of virtual reality education for dementia care was identified. In December 2021, a preliminary search of Google Scholar, limited to papers published after 2017, using the search string implement* virtual reality in higher education was also conducted. The preliminary search yielded a total of 17,200 hits. We screened the first 50 hits to identify papers on the implementation of virtual reality in higher education, and 9 articles were considered relevant for full-text reading.

We identified 3 reviews on virtual reality in higher education, one of which reported on virtual reality in higher health professional education [7], which included studies from 1990 to 2017 and found that most assessed the effectiveness of nonimmersive virtual reality. Virtual reality interventions with greater interactivity seemed to improve students’ competencies more than interventions with less interactivity. The review [7] concludes that immersive scenarios could make education programs more efficient and attractive. A review [1] of virtual reality in science and technology education found that there are problems pertaining to training programs, such as students finding virtual reality implementations to be unrealistic, due to the limited time and resources available to the students. Moreover, most virtual reality projects included in the review [1] were at experimental stages. A more recent review [5] of virtual reality in general higher education found that there were few design-oriented studies wherein the virtual reality apps were constructed based on a specific learning theory. Moreover, few papers included in the review [5] thoroughly described how virtual reality–based teaching can be adopted in the teaching curriculum, which is a central aspect of implementation of virtual reality in higher education. Fernandez [10] suggests that teacher technological competency is a barrier to successful implementation of virtual reality in education, and training in the use of the technology and in the pedagogical purposes of virtual reality are equally important [10].

Our preliminary findings—virtual reality’s rapidly changing technology nature and continued interest in virtual reality—indicate that a review on the implementation of virtual reality in higher health professional education would be timely. Because the use of virtual reality is still a novelty in higher education, descriptions and evaluations of their implementation in this setting are also likely to be few in number; therefore, a scoping review was considered the most appropriate review methodology because its purpose is to provide an overview of available evidence [11].

### Aim

The aim of this scoping review is to identify studies reporting on implementation of virtual reality in higher health professional education, to identify barriers and facilitators for implementation, and to highlight research gaps in this area.

## Methods

### Overview

The scoping review will be conducted according to JBI Evidence Synthesis methodologies [11-13] and reported according to the PRISMA-ScR (Preferred Reporting Items for Systematic Reviews and Meta-Analyses Extension for Scoping Reviews) checklist [14].

### Search Strategy

CINAHL, Academic Search Elite, Education Source, and Google Scholar will be searched. Manual searching of key items, reference tracking, and citation tracking will also be performed. We will also search for white papers manually.

Initial searches were conducted by SSL and a university librarian in November and December 2021 to refine the search string for electronic databases (Table 1). Search terms, such as *Virtual reality*, *Higher Education (health)*, and *Implementation*, as well as several synonyms, were chosen (Table 1). The search words will be combined with the operator *AND*. Search criteria will be papers published from 2017 to 2022 and papers written in English (on higher education or health professional education, including medicine and continuing education with individuals over 18 years and on virtual reality or computer simulation).

Forward and backward citation searching will be conducted on papers that meet search criteria. We will also conduct manual searches on Norwegian government webpages to identify government reports, policy documents, and other material relevant to this scoping study.

**Table 1:** Search strategy development for Academic Search Elite.

Search string number	Query	Results
S10	S3 AND S6 AND S9 (Limiters - Published Date: 20170101-20221231. Narrow by Language: - english)	48
S9	S7 OR S8	950,876
S8	Implementation OR “program implementation”	437,365
S7	implement* or “implementation science” or “implementation method” or “implementation strateg*” or “program implementation” or “training programs”	950,876
S6	S4 OR S5	502,010
S5	“Medical education” or “higher education”	474,913
S4	“higher education (health)” or “nursing school” or “health scienc* educat*” or “allied health education” or “medical educat*”, “social science* educat*” or “healthcare education” or “health occupation students”	31,052
S3	S1 OR S2	21,497
S2	TI “virtual reality” OR AB “virtual reality” OR TI “VR” OR AB “VR”	21,436
S1	“Virtual reality in higher education” or “simulation methods and models”	79

### Study Selection Criteria

#### Participants

Studies with faculty or students in health-related fields (such as medicine, nursing, physiotherapy, occupational therapy, social education, disability nursing, dental care, pharmacy, and psychology) will be included.

#### Concept

The primary concept under investigation in this review is the implementation of virtual reality, with implementation defined as “the act of putting a plan into action or starting to use something” [15]. We will explore barriers and facilitators for the implementation of virtual reality. We define virtual reality as a digital representation of a 3D environment, presented in a head-mounted display for a fully immersive experience [1,2]. Based on the above, papers addressing virtual reality implementation will be included.

#### Context

Only papers on virtual reality implementation in higher health professional education (Bachelor’s and Master’s programs) or continuing education for health care professionals will be included. Continuing education was deemed to be relevant because its aim is to provide secure lifelong learning for health care professionals [16].

#### Types of Sources

We will consider quantitative, qualitative, and mixed methods study designs for inclusion. In addition, reviews, background papers, and white papers will be considered for inclusion to gain a broad understanding of the topic at hand.

#### Exclusions

Papers describing the implementation of virtual reality in clinical use for patients or children will also be excluded.

### Study Selection Process

Identified records will be uploaded into EndNote (version 20; Clarivate Analytics), and duplicates will be removed. Titles and abstracts will be screened by 4 independent reviewers using Rayyan’s (Qatar Computing Research Institute) [17] blinded screening functionality. We will meet and discuss the screening process several times to ensure consistency. For studies that meet selection criteria, the full-text papers will be assessed by all 5 reviewers. Reasons for the exclusion of full-text papers will be recorded and reported. Disagreements between the reviewers at any stage of the selection process will be resolved by discussion within the research group.

### Data Extraction and Analysis

**Figure 1:**
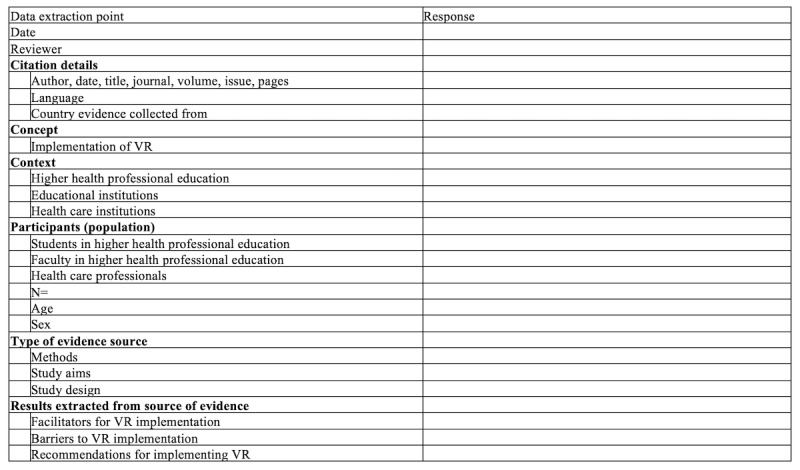
Data extraction tool. VR: virtual reality.

Data will be extracted from papers selected for inclusion in the scoping review independently by all authors. We will use an extraction instrument (Figure 1) adapted from JBI guidelines [11]. The draft was piloted on 1 paper that met selection criteria to ensure that all members of the research team had a similar understanding of the items. This resulted in the team choosing to focus on only the 3 factors under the heading *Results extracted from source of evidence*.

The data extraction tool may be further modified and revised as necessary during the process of extracting data from each paper; such modifications will be recorded and reported. Given that the aim is to map available evidence, critical quality appraisal of the papers will not be performed [11]. Qualitative content analysis will be used to identify key themes.

## Results

The project is ongoing. Searches were conducted in January 2022 in Academic Search Elite, Education Source, CINAHL, and Google Scholar. The review is expected to be completed by fall 2022, after which time it will be submitted for publication.

## Discussion

We expect to abstract 5 to 8 themes that present the challenges of and recommendations for implementation of virtual reality initiatives in health professional education programs; and recommendations for future research. We will discuss principal findings, prior work, strengths and limitations, and future directions. In part, the findings of the review will be used to inform the implementation of a virtual reality–based educational program that is currently being developed in a private higher educational institution in Norway for undergraduate programs in nursing, occupational therapy, social education, and social work.

Limitations of this scoping review protocol are that relevant sources of information may be omitted by our choice of search words and our choice of languages. Moreover, we will not rate the quality of evidence, and therefore, implications for practice cannot be assessed [11]. Since use of virtual reality in health professional education is a novelty, we may face some challenges in the literature search process in identifying literature specifically on implementation. However, since our preliminary searches yielded papers on virtual reality in health professional education, we believe that this scoping review is timely and relevant.

## Abbreviations

PRISMA-ScR: Preferred Reporting Items for Systematic Reviews and Meta-Analyses Extension for Scoping Reviews
